# Plant Phenotyping: Past, Present, and Future

**DOI:** 10.34133/2019/7507131

**Published:** 2019-03-26

**Authors:** Roland Pieruschka, Uli Schurr

**Affiliations:** IBG-2 Plant Sciences, Forschungszentrum Jülich GmbH, D-52425 Jülich, Germany

## Abstract

A plant develops the dynamic phenotypes from the interaction of the plant with the environment. Understanding these processes that span plant's lifetime in a permanently changing environment is essential for the advancement of basic plant science and its translation into application including breeding and crop management. The plant research community was thus confronted with the need to accurately measure diverse traits of an increasingly large number of plants to help plants to adapt to resource-limiting environment and low-input agriculture. In this overview, we outline the development of plant phenotyping as a multidisciplinary field. We sketch the technological advancement that laid the foundation for the development of phenotyping centers and evaluate the upcoming challenges for further advancement of plant phenotyping specifically with respect to standardization of data acquisition and reusability. Finally, we describe the development of the plant phenotyping community as an essential step to integrate the community and effectively use the emerging synergies.

## 1. Introduction

Physics and chemistry are quantitative sciences providing the opportunity to develop predictive models, which were the key to many developments in the last century and have been profoundly changing and effecting our lives (e.g., [1]). In biology, the development of sequencing techniques has generated the opportunity to analyze the genome, which provides the organism with the basic toolbox to grow and to reproduce in its dynamic life-long environment. The sequencing technology combined with data analysis of genome has revolutionized our understanding of biology in the last two decades [2, 3] and—in many cases today—may allow us to predict the performance based on the genetic constitution of an organism [4].

However, the phenotype—developed from the interaction of the organism with its environment from its very early stages until the end of its life—poses numerous additional challenges for the quantitative analysis [5]. It has long been an almost impossible task to record both biological system and its environment in the multitude of spatial and temporal dimensions that are necessary to understand the development of a specific phenotype. This is not only because biology handles many species that have very diverse habitus and designs, in contrast to, e.g., medicine, which “only” considers a single species with very limited variability in comparison with, e.g., plant species. In addition, the plasticity of phenotypes—specifically in plants—is enormous and part of their adaptation to the given environments. This plasticity of the phenotype even generates feedback loops: e.g., plants exposed to a limiting environment (e.g., drought) stay (generally) smaller and often adapt their structure (shoot and root) and their physiology. This makes them more resistant against the next limitation. Thus, plasticity of the phenotype in response to the environment often has consequences for the environmental exposure of the plant at later stage of its life. This “memory effect” makes phenotypes very dependent on previous experiences of the crop and their adaptation to it [6]. This even can include impact across generations, when environmental cues that affected mother plants during seed filling cause epigenetic or maternal effects on the seed composition and quality [7].

Other challenges originate from the need for deep mechanistic understanding of the interaction between a plant and local conditions. This might require deep phenotyping methods with high spatial and temporal resolution of very specific traits that allow the assessment of the dynamic nature of adaptation to the local conditions, e.g., dynamics of carbon allocation to plant organs [8] or the patterns of root and shoot growth [9]. Phenotypes are determined from essentially all parts of plants and from entire plant assemblies, which are interesting to science to understand basic processes determining plant performance or many practical applications in breeding, crop management, or even processing harvested crop parts. Therefore, phenotyping technologies and protocols are important tools that are needed, e.g., to assess the structure and function of seeds, roots and root systems, storage organs above- and belowground, leaves, and entire canopies, as well as fruits, flowers, and many more. This raises challenges with respect to throughput, sensitivity, and accuracy. Often trade-offs need to be accepted to address the major limiting factor of a specific application.

Plant phenotyping is an important tool to address and understand plant environment interaction and its translation into application in crop management practices [10], effects of biostimulants [11], microbial communities [12], etc. The plant breeding process is one of the major applications of phenotyping in practice today and many developments in plant phenotyping are governed by breeding targets [13]. Here novel technologies must provide advantages with respect to levels of throughput, applicability under field conditions (at many diverse weather conditions), and specifically the usefulness in the breeding process itself, the heritability of the determined traits.

Novel opportunities to address these challenges have been developed specifically during the last two decades or so through novel sensors, automation, and quantitative data analysis. New developments in sensor technology for plant phenotyping have led to a substantial increase in what could be analyzed and at which capacity and throughput [14]. These technologies opened new opportunities to phenotype a large diversity of plants ranging from model plants to crops and forests across scales from cellular to tissue and organ level, from single plants in a greenhouse to canopies and vast agricultural areas, and to address time scales from nanoseconds, e.g., during light conversion in photosynthetic processes to extended seasonal development. All of these became possible in countless environmental scenarios resulting in increasing amounts of data posing the challenge to explore these large datasets, e.g., to analyze plant genomes and link them to plant phenotypes. The availability and reuse of interoperable data are here in focus and require standardization from calibration procedures of the sensors, through standard operation procedures of experiments and extraction of data from multimodal images to experimental design and metadata to data ontology formats and standardized interfaces to generate meaningful data. To further advance the development, a close interaction between groups from plant scientists and geneticist to hardware and software engineers as well as data managers was and is still required. Such joint efforts initially were possible in specialized institutes and multidisciplinary working groups. Today, national and international networks and initiatives play an ever-increasing role and provide a nucleus linking different competences that are needed. This made plant phenotyping in the past decade a pivotal research area in basic and applied plant sciences, being attractive and visible to scientists, users, and the public. It has initiated a dialogue among scientists, breeders, agricultural industry, funding agencies, policy makers, and the public and has advanced our understanding of plant environment interaction, which is central to address future grand challenges.

## 2. Noninvasive Plant Measurements

Plant processes such as growth, photosynthesis, and transpiration or yield formation are the basis of life on our planet, nourishing the presently 7 billion people. These processes will have to be further improved to achieve the goal of feeding the increasing population, at less footprint, on less land, and with less water, in a changing climate with increasing demand for quantity and quality of food and nonfood products from plants [15]. The process of intended modification of plant properties such as yield has been initiated over ten thousand years ago when people started to domesticate plants for human uses. The fact that such phenotypic selections can lead to a persistent improvement of crops, which is stable beyond generation, was the fundament of breeding from the early beginning of domestication of crops and fundamentally altered the course of human history [16]. The adaptation of plants to crop cultivation was vital to the shift from hunter-gatherer to agricultural societies, and it stimulated the development of cities and modern civilizations. Early farmers selected food plants with desirable characteristics and used these as a seed source for subsequent generations, resulting in an accumulation of characteristics over time. At that time, phenotyping was the only option to select through experience, since the basic principles of genetics have only been determined in relatively recent times. Mendel's heritability laws discovered over a hundred years ago ultimately led to the establishment of genetics and the basis for the understanding of inheritance, which is still the basis in modern plant breeding. Measurement of diverse plant properties such as structure and function has been often performed by the collector's eyes, manually or invasively, i.e., using destructive methods. Development of first sensors or tools to assess plant properties in response to external stimuli has been addressed by the pioneers in plant sciences in 19^th^ century, e.g., by Pfeffer's development of an apparatus for plant growth measurement [17]. A description of historic methods for analyzing different traits has been summarized by Ruge [18].

Within the last decades, several sensors and computer vision tools have been developed and became pivotal for quantifying plant traits with increasing throughput and accuracy [14]. Imaging based systems to measure plant traits were increasingly used starting in the 1990s following the development of charge-coupled-device (CCD) and complementary-metal–oxide–semiconductor (CMOS) technologies [19]. The measurements often focused on measuring just single organs, e.g., by measuring growth of leaves or roots [9, 20]. Further development has led to 3D shoot imaging approaches [21] for functional imaging of photosynthesis and transpiration [22, 23] and transport processes within a plant [8]. Application of noninvasive technology for plant phenotyping in high throughput started with the model plant* Arabidopsis* [24] with sufficient number of genotypes providing the opportunity to associate traits to the genetic make-up of a plant. In recent years phenotyping using noninvasive technologies has achieved strong development, including detailed analysis as well as high-throughput installations, environmental simulation facilities, greenhouses, and field phenotyping systems. Modern installations include high-resolution phenotyping used in tomographic measurements (e.g., Magnetic Resonance Imaging (MRI), Positron Emission Tomography (PET), and Computer Tomography (CT) or even combination of these) and high-throughput systems in small, medium, and large facilities simulating environmental conditions in closed buildings or in greenhouse conditions. Intensive field sites provide opportunities to analyze crop performance in the field in depth, partly even allowing simulation of future climates and CO_2_-conditions. Increasingly, mobile and higher throughput field systems, e.g., by using unmanned aerial vehicles (UAVs) with a variety of diverse sensors, become available and allow breeders to monitor genotype performance in breeding plots and farmers to utilize novel technologies for precision crop management. Further advancement of plant phenotyping can benefit from developments in disciplines such as remote sensing, robotics, computer vision, and artificial intelligence [14, 25, 26].

## 3. Data Interoperability and Standardization

Linking sensor technology, i.e., data acquisition, with the prospect to reuse the acquired data is the essential next step that may further define sensor development and requires the interaction of the relevant stakeholders.

One goal of modern phenomics is the integration of data into structured and searchable databases following the FAIR principle (findable, available, identifiable, reusable) to enhance the ability of machines to automatically find data and thus enable the reuse of these data by individuals [27]. Multidimensional data are essential for the understanding of the mechanisms governing basic processes in life and plant sciences. A key issue in this context is the quality of the data, considering the entire data acquisition pipeline in plant phenotyping. This includes metadata with clear measurement protocols that need to start with the sensor calibration, the measurement description, and analysis of the often obtained images with the respective algorithms [28]. Visualization of data may further improve the data quality by providing a tool for reporting and interpretation, specifically for quality control of these data [29]. In the multidimensional plant phenotyping context, assessment of detailed environmental conditions represents another key element for the interpretation of the interaction of a genotype with the environment. For example, the conditions for a single plant may vary even within controlled growth facilities [30], and genotypes that perform well in controlled environments do not necessarily perform best in the field [31]. This is particularly important when plants are tested under suboptimal conditions, such as a low nutrient or water supply [32]. Thus, a comprehensive report of environmental conditions during experiments is of importance under controlled and field conditions. To make data reusable within the plant science community the MIAPPE (Minimal Information about Plant Phenotyping Experiments) recommendations have been developed and are continuously updated to allow a description of all necessary metadata, including the environment [33, 34].

There has been substantial progress in data management in plant phenotyping (for recent reviews see [29, 35]). However, quite often the complex phenotyping data remain unavailable because there is a lack of infrastructure to store and handle the data and lack of standardized formatting. Data semantics and automated approaches are often difficult and prone to errors in dealing with the increasing amount of data. Thus, a close interaction between data managers and generators is indispensable to further advance the development of information systems that allow reusability of data, and consider all legal, security, and privacy aspects.

## 4. Demand for Community Integration

In order to generate enough interaction and critical mass to effectively use the existing synergies in plant phenotyping, several national plant phenotyping infrastructures were established. One of the first institutionalized infrastructures was the Australian Plant Phenotyping Facility (APPF, https://www.plantphenomics.org.au/) with its three nodes in Canberra (CSIRO and ANU) and in Adelaide. Since the early start, the installations expanded beyond the in-house systems and the Plant Accelerator to field phenotyping stations and installations across Australia. The German Plant Phenotyping Network (DPPN, https://dppn.plant-phenotyping-network.de/index.php?index=6) was started in 2012, followed by PHENOME (France, https://www.phenome-emphasis.fr/phenome_eng/) in 2013. These nationally funded projects focused on investment in large-scale infrastructures, data analysis systems, and access to the infrastructure in a coordinated manner. In other countries, phenotyping infrastructures were established in a less coordinated manner by individual project funding and networking activities (e.g., UK, Belgium, Finland, Denmark, US, Canada, Brazil, and Argentina). Recently, more national and regional projects and institutions have been launched in China and by the CGIAR centers within the Excellence in Breeding Platform (http://excellenceinbreeding.org/). Crop-specific initiatives, like the Wheat Initiative (http://www.wheatinitiative.org/) of the G20, generated crop-specific networks to exchange knowledge and expertise in crop phenotyping.

Because the plant phenotyping landscape has developed substantially and the demand is ever increasing, a number of projects have been initiated to utilize and further develop the phenotyping infrastructures. In Europe, plant phenotyping projects to address* Arabidopsis* phenotyping (AGRON-OMICS, https://cordis.europa.eu/project/rcn/85221_en.html) and to develop technology for phenotyping of different crops and plant organs (SPICY, http://www.spicyweb.eu/; EURooT, http://www.euroot.eu/; DROPS, https://www6.inra.fr/dropsproject) paved the way to the EU funded FP7 I3 project EPPN (2012-2015 European Plant Phenotyping Network, http://www.plant-phenotyping-network.eu/). EPPN as a starting community project provided transnational access to 23 experimental plant phenotyping installations across Europe. The project has resulted in 66 transnational access experiments and more than 50 peer reviewed publications. Based on the success of EPPN and the increasing demand for plant phenotyping, a H2020 advanced community I3 project, EPPN2020, was approved (2017-2021, https://eppn2020.plant-phenotyping.eu/), providing the opportunity for ~200 potential plant phenotyping transnational access experiments and integrating 31 key plant phenotyping installations in 11 European countries. Furthermore, the COST Action “The quest for tolerant varieties - Phenotyping at plant and cellular level” started in 2011 and created a network and very intense interaction of European scientists with expertise on phenotyping, various omics areas, and plant physiology, which is important to understand tolerance (http://www.cost.eu/COST_Actions/fa/FA1306). In the USA, the Genome to Fields Initiative (https://www.genomes2fields.org/) has developed an extensive multistate, multiyear dataset of corn phenotype data from a network of field phenotyping sites. The Nord American Plant Phenotyping Network (http://nappn.plant-phenotyping.org/) is currently an important initiative to link scientists and researchers in the rapidly evolving plant phenotyping area in northern America while the LatPPN [36] is aimed at integrating the Latin American countries.

**Figure 1:**
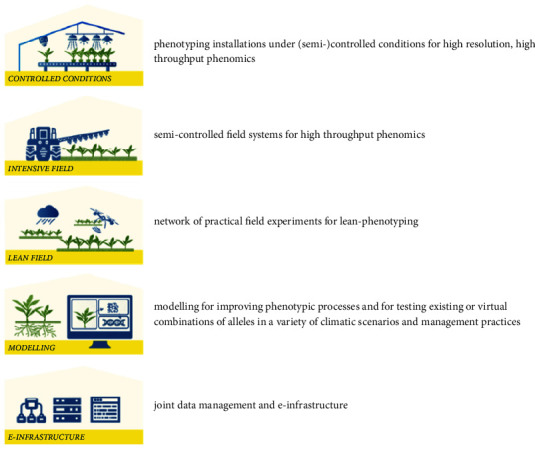
Five EMPHASIS infrastructure pillars to comprehensively address plant phenotyping.

Many initiatives formed a strong basis for international cooperation: the most prominent was the EMPHASIS project that was set up to install a European Infrastructure for Multiscale Plant Phenotyping and Simulation for Food Security in a Changing Climate (EMPHASIS, https://emphasis.plant-phenotyping.eu/) based on the international mechanism of the European Strategy Forum for Research Infrastructure (ESFRI, https://www.esfri.eu/). EMPHASIS has been listed on the ESFRI roadmap in 2016 within the Health and Food strategy. Since entering the ESFRI roadmap, EMPHASIS could already support (1) the acquisition of funding for plant phenotyping infrastructure via national/regional infrastructure funding mechanisms (NordPlant in Scandinavia, NPEC in The Netherlands, NaPPI in Finland), (2) proposals for the integration of plant phenotyping infrastructure on national infrastructure roadmaps (Estonia, Poland), (3) the establishment of national plant phenotyping initiatives (Ireland, Italy, Austria, Czech Republic, Cyprus), and (4) the initiation of national plant phenotyping initiatives (Bulgaria, Denmark, Greece, Israel, Norway, Portugal, Romania, Serbia, Slovakia, Spain, Sweden, Switzerland). EMPHASIS aims to develop and provide access to facilities that address multiscale plant phenotyping in different agroclimatic scenarios in Europe and centers around five different infrastructure pillars that will allow comprehensively addressing the diversity of traits contributing to plant performance in diverse environmental scenarios and enabling predictions making (Figure 1).

On a global scale, the International Plant Phenotyping Network (IPPN, https://www.plant-phenotyping.org/) was founded by leading research institutions to promote and educate state-of-the-art plant phenotyping in 2016. Today, nearly 40 organizations collaborate in organizing major symposia and workshops in relevant domains of plant phenotyping at an international scale. They run workgroups on highly relevant and emerging topics including imaging and image analysis, root phenotyping, seed phenotyping, approaches for affordable phenotyping, phenotyping under controlled environment, data management, and sensor and technology for phenotyping. The IPPN also strives to generate interactions between academia and industry with its recent decision to open up to industry members, offering opportunities for industry-based users and developers of phenotyping technologies.

All these projects and initiatives have considerably changed the landscape of phenotyping infrastructures addressing key questions in plant sciences and integrating the community globally. A recent analysis in the last few decades based on bibliometric mapping pictures plant phenotyping as a rapidly growing research area [37, 38]. The resulting demand for phenotyping with new technology and automation has also led to the establishment of a growing commercial sector with focus on plant phenotyping (https://www.datamintelligence.com/research-report/plant-phenotyping-market/?gclid=EAIaIQobChMI09idzIul4AIV6LDtCh3bnQFAEAAYASAAEgKT_fD_BwE). This growing plant phenotyping landscape has generated the opportunity and the demand for coordinated data generation, management, analysis, and integration across platforms, measurements, and experiments. This will allow us to link phenotypic and genetic approaches to characterize and finally improve a wide range of crops, but also use these modern approaches to improve scientific understanding of plant performance and ecological adaptation in an ever-changing environment. Specifically, modelling and data management are important tools that need to be integrated in and/or interface with phenotyping pipelines from data acquisition to reuse based on data formats and common semantics enabling automated analysis of the growing amounts of data. Since the plant phenotyping space is too immense to address all genotypes under all relevant environmental conditions, modelling based on FAIR data to further develop and validate models is pivotal in predicting phenotypic results of traits and genetic variation in target environments, essentially linking all pillars in Figure 1.

Plant phenotyping has rapidly emerged in the past decades and generated many new opportunities addressing the diverse demands, in which phenotyping is needed. However, the development is still evolving rapidly. We still need to generate more capacity, implement the new technologies seamlessly into the workflow of users in breeding and academia, develop proper access opportunities, and establish data management systems that allow data exchange and information gain across installations, locations, and experiments. This needs to be done in parallel with continued implementation of novel technologies. Plant phenotyping has come a long way, but there are still many challenges and opportunities ahead.
